# Association between transarterial chemoembolization refractoriness and prognosis in Chinese patients with hepatocellular carcinoma: a large retrospective cohort study

**DOI:** 10.3389/fonc.2024.1483949

**Published:** 2025-01-20

**Authors:** Qinxue Sun, Ziliang Wu, Xi Yin, Feng Li, Ri Liu

**Affiliations:** ^1^ Department of Interventional Radiology, Ningbo Medical Center LiHuiLi Hospital (The Affiliated LiHuiLi Hospital of Ningbo University), Ningbo, Zhejiang, China; ^2^ Department of Radiology, Mental Health Institute of Inner Mongolia Autonomous Region (The Third Hospital of Inner Mongolia Autonomous Region, Brain Hospital of Inner Mongolia Autonomous Region), Hohhot, Inner Mongolia, China

**Keywords:** hepatocellular carcinoma, transarterial chemoembolization, TACE refractoriness, overall survival, propensity score matching

## Abstract

**Background:**

Hepatocellular carcinoma (HCC) is commonly treated with transarterial chemoembolization (TACE) in intermediate stages. Existing international definitions of TACE refractoriness may not fully suit Chinese patients. The Chinese College of Interventionalists (CCI) proposed a tailored definition, but its impact on HCC prognosis is still limited.

**Methods:**

This study included 844 patients with Barcelona Clinic Liver Cancer (BCLC) stage B HCC from a multicenter dataset. Propensity score matching (PSM) was used to minimize baseline differences between the TACE-Refractoriness (n = 54) and TACE-Non-Refractoriness (n = 108) groups. Kaplan-Meier survival analysis and multivariate Cox regression models were performed to evaluate the association between TACE-Refractoriness and OS. Subgroup analyses were conducted across key clinical and tumor-related characteristics.

**Results:**

Kaplan-Meier survival analysis indicated that patients classified as TACE-Refractory exhibited significantly shorter OS compared to those categorized as TACE-Non-Refractory in both the original and matched cohorts (*P* < 0.001). Furthermore, multivariate analysis identified TACE refractoriness as a significant predictor of poorer OS, yielding an adjusted hazard ratio (HR) of 5.96 (95% CI: 3.39-10.5, *P* < 0.001). Subgroup analysis further demonstrated the robustness of these findings across subgroups, except in female patients (HR = 3.0, 95% CI: 0.72–12.52; *P*=0.131).

**Conclusions:**

CCI-defined TACE refractoriness is associated with reduced OS in patients with BCLC stage B HCC undergoing TACE.

## 1 Introduction

Hepatocellular carcinoma (HCC) is one of the most prevalent malignancies worldwide and remains a leading cause of cancer-related mortality (1, 2). In China, it ranks as the second most common cause of cancer death, largely due to the high incidence of hepatitis B and C infections, which are primary risk factors (3). Despite advances in early detection and treatment, the majority of HCC cases are diagnosed at intermediate or advanced stage, where curative options such as surgical resection or liver transplantation are often no longer feasible (4). For patients with intermediate-stage HCC, transarterial chemoembolization (TACE) is the standard treatment, particularly for those classified as Barcelona Clinic Liver Cancer (BCLC) stage B (5). However, repeated TACE procedures can lead to cumulative liver damage and eventual treatment resistance, a condition known as TACE refractoriness (6, 7).

The concept of TACE refractoriness is crucial for optimizing HCC management, particularly in patients who initially respond to TACE but later exhibit disease progression despite continued treatment. Various definitions of TACE refractoriness have been proposed internationally, including those by the Japan Society of Hepatology (JSH) and the European Association for the Study of the Liver (EASL) (7, 8). However, these definitions often lack specificity for the Chinese population, where HCC has distinct epidemiological and clinical characteristics. In response, the Chinese College of Interventionalists (CCI) developed a definition tailored to Chinese patients, known as CCI-defined TACE refractoriness (9). This definition emphasizes the importance of standardized and refined TACE procedures and evaluates disease progression based on modified Response Evaluation Criteria in Solid Tumors (mRECIST) (10) criteria within 1-3 months after the third TACE session, providing a more precise framework for discontinuing ineffective TACE and transitioning to alternative therapies.

Using the CCI-defined TACE refractoriness criteria is particularly relevant in the Chinese context, where high tumor burden and advanced disease at diagnosis are common (9). Early identification of TACE refractoriness is crucial for preventing unnecessary liver damage and for guiding the timely initiation of alternative treatments, such as systemic therapy or clinical trial enrollment (11). This study aims to evaluate the impact of CCI-defined TACE refractoriness on overall survival (OS) in patients with HCC, using a propensity score-matched analysis to control for potential confounding factors. Understanding the prognostic implications of TACE refractoriness in this population will contribute to more effective treatment strategies and improve clinical outcomes for patients with HCC.

## 2 Patients and methods

### 2.1 Patient selection

This study is a secondary analysis based on a publicly available dataset from Shen et al (12). The original dataset comprised HCC patients diagnosed between January 2007 and December 2016 from multiple institutions, including the Sun Yat-sen University Cancer Center (SYSUCC) and three affiliated hospitals. The cohort included a total of 10,543 patients with newly diagnosed HCC who were retrospectively reviewed across three distinct sub-cohorts: derivation, internal testing, and multicenter testing cohorts.

Patients were included in the original study based on the following criteria (1): a confirmed diagnosis of BCLC stage B HCC; (2) availability of complete clinical and imaging data at the time of diagnosis, including abdominal computed tomography (CT) or magnetic resonance imaging (MRI), chest radiography or CT, routine bloodwork, biochemical tests, serum alpha-fetoprotein (AFP) levels, and coagulation profiles; and (3) no history of other malignancies. A total of 8,523 patients were excluded for not meeting these inclusion criteria. Detailed individual-level exclusion reasons for these patients were not available in the original dataset.

**Figure 1:**
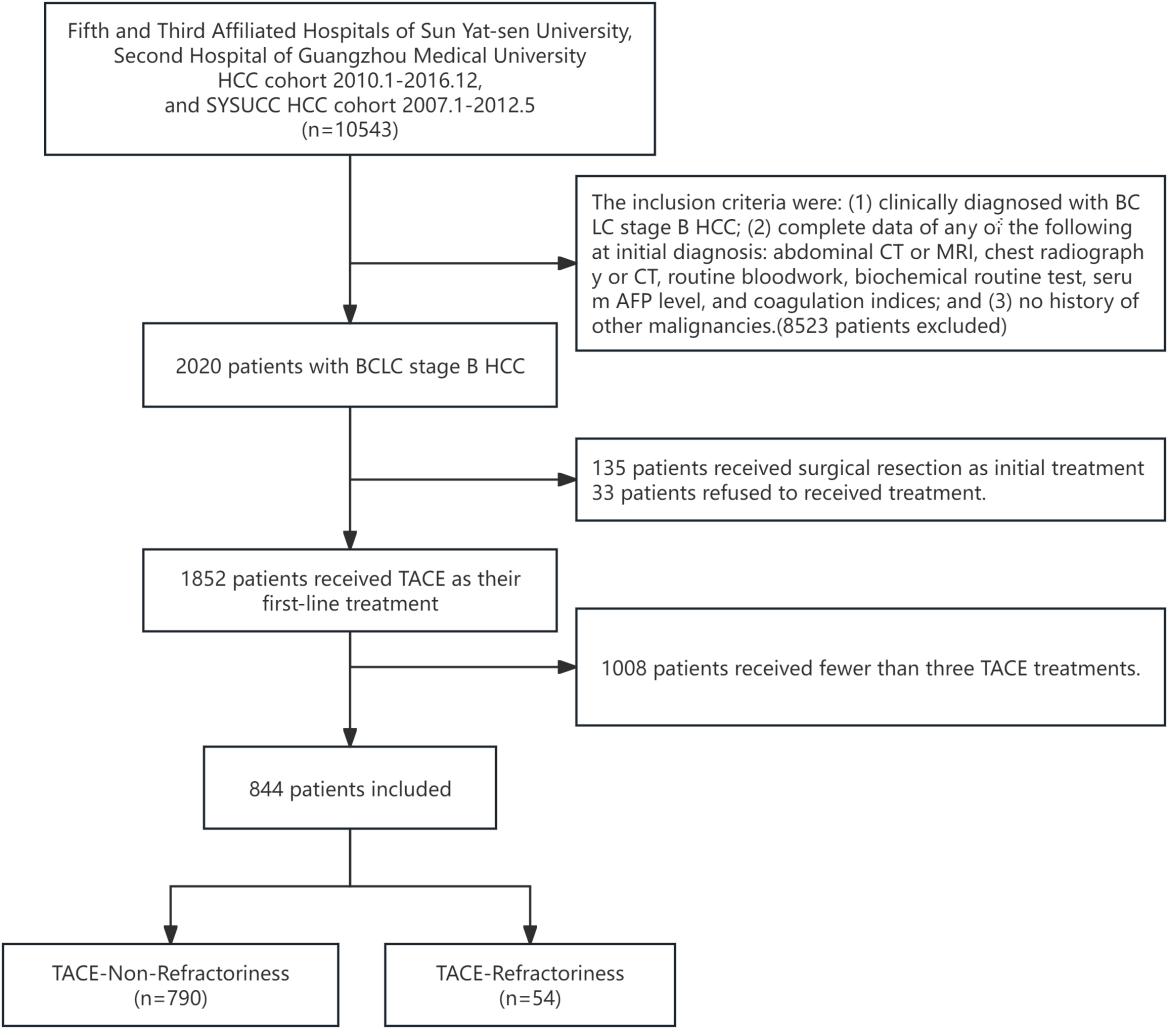
Flowchart of study design. A total of 10,543 HCC patients initially identified, 8,523 were excluded based on the following criteria: (1) lack of a clinical diagnosis of BCLC stage B HCC, (2) incomplete initial diagnostic data, and (3) history of other malignancies. This left 2,020 patients with BCLC stage B HCC. Subsequently, 135 patients who received surgical resection as initial treatment, 33 patients who refused treatment, and 1,008 patients who had received fewer than three TACE treatments were further excluded, resulting in a final study population of 844 patients. These patients were divided into two groups: TACE-Non-Refractoriness (n=790) and TACE-Refractoriness (n=54) based on the CCI criteria for TACE refractoriness.

For this study, we further refined the patient selection criteria to focus exclusively on BCLC stage B HCC patients who underwent at least three sessions of TACE treatment, as this threshold is critical for defining TACE refractoriness using the CCI criterial (9, 13). The final cohort included 844 patients, comprising 790 in the TACE-Non-Refractoriness group and 54 in the TACE-Refractoriness group before propensity score matching (PSM). The inclusion and exclusion process is summarized in the flowchart (**Figure 1**).

### 2.2 Ethical statement

The research protocol (2017-FXY-129) received approval from the relevant research department at SYSUCC (12), and ethical exemptions were granted by the Institutional Review Board (IRB) of Ningbo Medical Center LiHuiLi Hospital. This study is a secondary analysis based on pre-existing primary data that were anonymized to ensure privacy. As a result, the requirement for informed consent was waived. The study adhered to full compliance with the principles outlined in the Declaration of Helsinki (14).

### 2.3 TACE and follow-up

The TACE procedure was performed following a standardized protocol across all participating centers to ensure consistency. TACE involved selective or supra-selective catheterization of the tumor-feeding artery using a microcatheter. A mixture of lipiodol (5–20 mL) and chemotherapeutic agents, including doxorubicin (20–60 mg), was injected, followed by embolization with gelatin sponge particles (300–500 μm) or polyvinyl alcohol (PVA) particles (12). For patients with bilobar lesions, TACE was performed sequentially to minimize liver toxicity. The procedures were conducted by experienced interventional radiologists in accordance with the guidelines of the CCI and institutional protocols.

After the initial TACE session, patients were followed up every 4–6 weeks with contrast-enhanced CT or magnetic resonance imaging (MRI) to evaluate tumor response, along with liver function tests and serum AFP levels. Tumor response was assessed using the mRECIST criteria. For patients achieving complete remission, follow-ups were scheduled every 2–3 months during the first two years and every 3–6 months thereafter.

During follow-up visits, assessments included abdominal imaging through contrast-enhanced CT or MRI. Tumor response was evaluated using the mRECIST criteria. Decisions regarding further treatments, including additional TACE, ablation, or systemic therapy, were made by a multidisciplinary team based on the patient’s response and overall condition.

### 2.4 Data collection

Baseline data were collected from the hospital records prior to initial TACE, including demographic information, tumor characteristics (size, number, distribution), liver function parameters (albumin (ALB), aspartate transaminase (AST), prothrombin time (PT), total bilirubin (TBIL)), and serum AFP levels. Imaging studies were reviewed to assess treatment response according to the mRECIST criteria (10).

To address missing data in certain covariates (<5%), the fully conditional specification multiple imputation (FCS-MI) method was employed (15). Five imputed datasets were generated using this method. For subsequent analyses, one imputed dataset was randomly selected to perform all statistical modeling, ensuring consistency and reducing potential biases.

According to the 2021 CCI definition and expert consensus on TACE refractoriness (9), TACE refractoriness is defined as the progressive disease (PD) of the intrahepatic target lesion, compared to its status before the first TACE treatment, after receiving three or more standardized and refined TACE procedures. Notably, the progression of untreated intrahepatic lesions, new lesions, and extrahepatic metastases is not considered as TACE refractoriness.

### 2.5 Statistical analysis

Baseline characteristics were assessed using appropriate statistical tests: continuous variables were analyzed with t-tests or Mann-Whitney U tests depending on distribution, while categorical variables were compared using chi-square tests or Fisher’s exact tests. To minimize bias in baseline characteristics, PSM was performed with a 1:2 ratio using the nearest-neighbor method and a caliper of 0.1 to minimize baseline differences, and standardized mean differences (SMDs) were used to evaluate balance before and after matching, with SMD < 0.1 considered acceptable. OS rates were calculated using the Kaplan-Meier method. To determine the association between TACE-Refractoriness and OS in HCC patients, Multivariable Cox proportional hazards regression models were used to estimate hazard ratios (HRs) for OS, adjusting for potential confounders in three progressive models (16–18). Model 1 included demographic variables (age and sex). Model 2 extended Model 1 by adding tumor-related characteristics, including tumor size (<5 cm *vs*. ≥5 cm), tumor number (single *vs*. multiple), tumor distribution (unilobar *vs*. bilobar), and ascites (present *vs*. absent). Model 3 further incorporated initial liver function and tumor marker variables, including ALB, TBIL, AFP, and AST. Post-PSM analyses were based on Model 3 to ensure robust adjustment for confounding factors.

All statistical analyses, including descriptive statistics, Pearson’s chi-squared test, Kaplan-Meier survival analysis, and Cox regression, were performed using Free Statistics software version 1.9, which incorporates the R statistical software version 4.3.2 (https://www.R-project.org, R Foundation).

## 3 Results

### 3.1 Clinical characteristics

A total of 844 patients with BCLC stage B HCC were included in the final analysis following the application of inclusion and exclusion criteria, as summarized in **Figure 1**. The cohort consisted of 790 patients in the TACE-Non-Refractoriness group and 54 patients in the TACE-Refractoriness group before PSM. After 1:2 PSM, 162 patients were analyzed, comprising 108 in the TACE-Non-Refractoriness group and 54 in the TACE-Refractoriness group, with baseline characteristics detailed in **Table 1**.

**Table 1:** Baseline characteristics of patients with hepatocellular carcinoma in the original/matched data (N=844).

Variables	Original Data Set				Matched Data Set			
	TACE-Non-Refractoriness (n = 790)	TACE-Refractoriness (n = 54)	SMD	*P* Value	TACE-Non-Refractoriness (n = 108)	TACE-Refractoriness (n = 54)	SMD	*P* Value
Sex, n (%)			0.052	0.715			0.04	0.81
Male	537 (68)	38 (70.4)			74 (68.5)	38 (70.4)		
Female	253 (32)	16 (29.6)			34 (31.5)	16 (29.6)		
Age(years), Mean ± SD	52.8 ± 11.6	55.6 ± 11.1	0.242	0.091	54.2 ± 11.0	55.6 ± 11.1	0.125	0.455
Size of the largest tumor(mm), Mean ± SD	61.6 ± 30.9	52.8 ± 26.4	0.309	0.039	51.9 ± 30.9	52.8 ± 26.4	0.029	0.867
Number of tumors, n (%)			0.541	< 0.001			0.1	0.577
Solitary	418 (52.9)	42 (77.8)			88 (81.5)	42 (77.8)		
≥2	372 (47.1)	12 (22.2)			20 (18.5)	12 (22.2)		
Tumor distribution, n (%)			0.293	0.142			0.18	0.494
Unilobar	418 (52.9)	23 (42.6)			40 (37)	23 (42.6)		
Bilobar	372 (47.1)	31 (57.4)			68 (63)	31 (57.4)		
Ascites, n (%)			0.03	1			0.062	1
Absence	772 (97.7)	53 (98.1)			105 (97.2)	53 (98.1)		
Presence	18 (2.3)	1 (1.9)			3 (2.8)	1 (1.9)		
ALB(g/L), Mean ± SD	39.2 ± 5.3	39.9 ± 6.5	0.113	0.376	39.9 ± 5.0	39.9 ± 6.5	0.001	0.998
PT (seconds), Mean ± SD	12.2 ± 1.3	12.3 ± 1.0	0.113	0.47	12.3 ± 1.2	12.3 ± 1.0	0.035	0.837
AFP (ng/ml), Median (IQR)	162.6 (11.0, 1926.8)	163.7 (28.9, 1874.5)	0.213	0.594	221.9 (23.0, 1184.0)	163.7 (28.9, 1874.5)	0.051	0.827
AST (U/L), Median (IQR)	67.0 (38.0, 152.8)	57.0 (40.6, 78.9)	0.184	0.167	57.0 (36.2, 160.5)	57.0 (40.6, 78.9)	0.002	0.635
TBIL (umol/L), Median (IQR)	18.9 (12.9, 27.3)	18.2 (14.3, 26.2)	0.104	0.995	20.2 (12.9, 24.3)	18.2 (14.3, 26.2)	0.028	0.929

SMD, standardized mean difference; ALB, albumin; PT, prothrombin time; AFP, Alpha-fetoprotein; AST, aspartate transaminase; TBIL, total bilirubin; SD, standard deviation.

Before PSM, significant baseline imbalances were observed between the two groups. For example, tumor number and tumor size showed large SMDs of 0.541 and 0.309, respectively. After PSM, the balance was improved for most variables, with SMDs reduced to acceptable levels (<0.1) for key covariates, including largest tumor size (SMD = 0.029) and alpha-fetoprotein (AFP, SMD = 0.051). However, slight residual imbalances remained for age (SMD = 0.125), tumor number (SMD = 0.1), and tumor distribution (SMD = 0.18).

Clinical parameters such as ALB and PT showed no significant differences between the TACE-Non-Refractoriness and TACE-Refractoriness groups in both the original and matched datasets. In the original dataset, P-values for ALB and PT were 0.37 and 0.47, respectively (t-test), while in the matched dataset, the corresponding P-values were 0.998 and 0.837 (t-test). For TBIL, no significant differences were observed in both datasets, with P = 0.99 in the original dataset (Mann-Whitney U test) and P = 0.929 in the matched dataset (Mann-Whitney U test).

### 3.2 Survival analysis of OS

**Figure 2:**
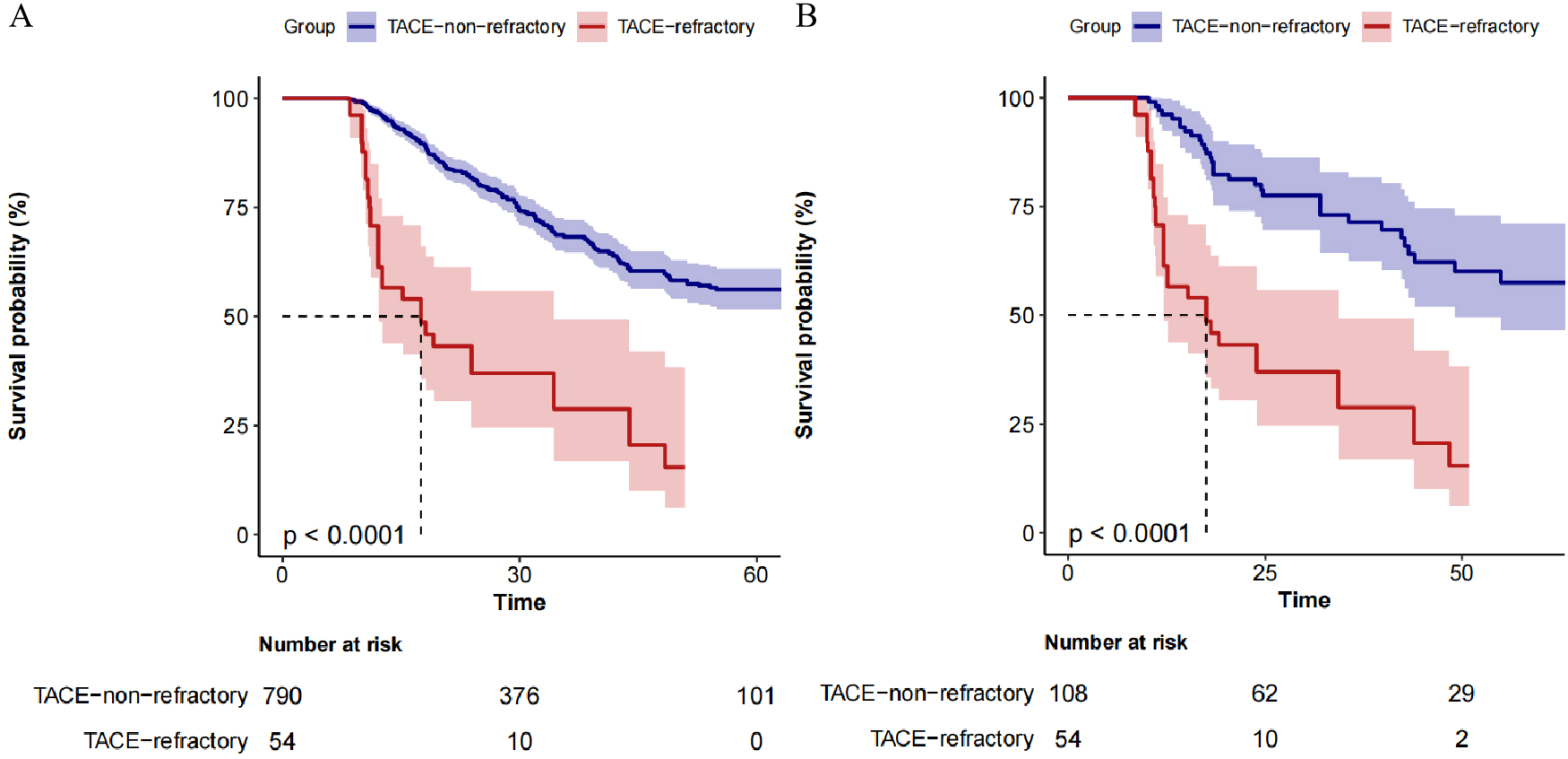
Kaplan-Meier Survival Curves of patients with BCLC stage B HCC. **(A)** depicts the survival of the entire cohort (N=844), revealing significantly poorer survival in the TACE-Refractory group (*P* < 0.001). **(B)** shows the survival in the matched cohort (n=162), where TACE-Refractoriness continued to predict worse outcomes (*P* < 0.001). These results highlight the adverse impact of TACE refractoriness on survival in patients with hepatocellular carcinoma.

The Kaplan-Meier survival curves for OS between the TACE-Non-Refractoriness and TACE-Refractoriness groups are shown in **Figure 2**. In the original dataset, patients in the TACE-Refractoriness group exhibited significantly reduced OS compared to those in the TACE-Non-Refractoriness group (P < 0.0001) (**Figure 2A**). After PSM, the survival disadvantage in the TACE-Refractoriness group persisted, as shown in the matched Kaplan-Meier curves (**Figure 2B**), with a significant difference in OS between the two groups (P < 0.0001).

### 3.3 Multivariate analysis of OS

Multivariate Cox regression analysis was conducted to evaluate the association between TACE-Refractoriness and OS. In the unadjusted model, TACE-Refractoriness was associated with an increased risk of death, with a hazard ratio (HR) of 4.1 (95% CI: 2.5–6.74; P < 0.001). After adjusting for age, sex, ascites, tumor characteristics (size, number, distribution), and liver function parameters (ALB, AFP, AST, and TBIL), the association remained significant, with an adjusted HR of 5.96 (95% CI: 3.39–10.5; P < 0.001). The stepwise inclusion of additional covariates across the models resulted in consistent increases in the estimated HR (**Table 2**).

**Table 2:** Association between TACE-refractoriness and TACE-non-refractoriness for overall survival using an extended model approach after propensity score matching.

	overall survival		
	HR	95%CI	p-value
Nonadjusted	4.1	2.5~6.74	<0.001
model 1	4	2.43~6.59	<0.001
model 2	5.05	2.98~8.56	<0.001
model 3	5.96	3.39~10.5	<0.001

Model 1 adjusts for age and sex; model 2 adjusts for model 1 + ascites, size, number and distribution of tumors, model 3 adjusts for model 2 + initial ALB, AFP, AST, TBIL. HR, hazard ratio; CI, confidence interval; ALB, albumin; AFP, Alpha-fetoprotein; AST, aspartate transaminase; TBIL, total bilirubin.

### 3.4 Subgroup analysis

**Figure 3:**
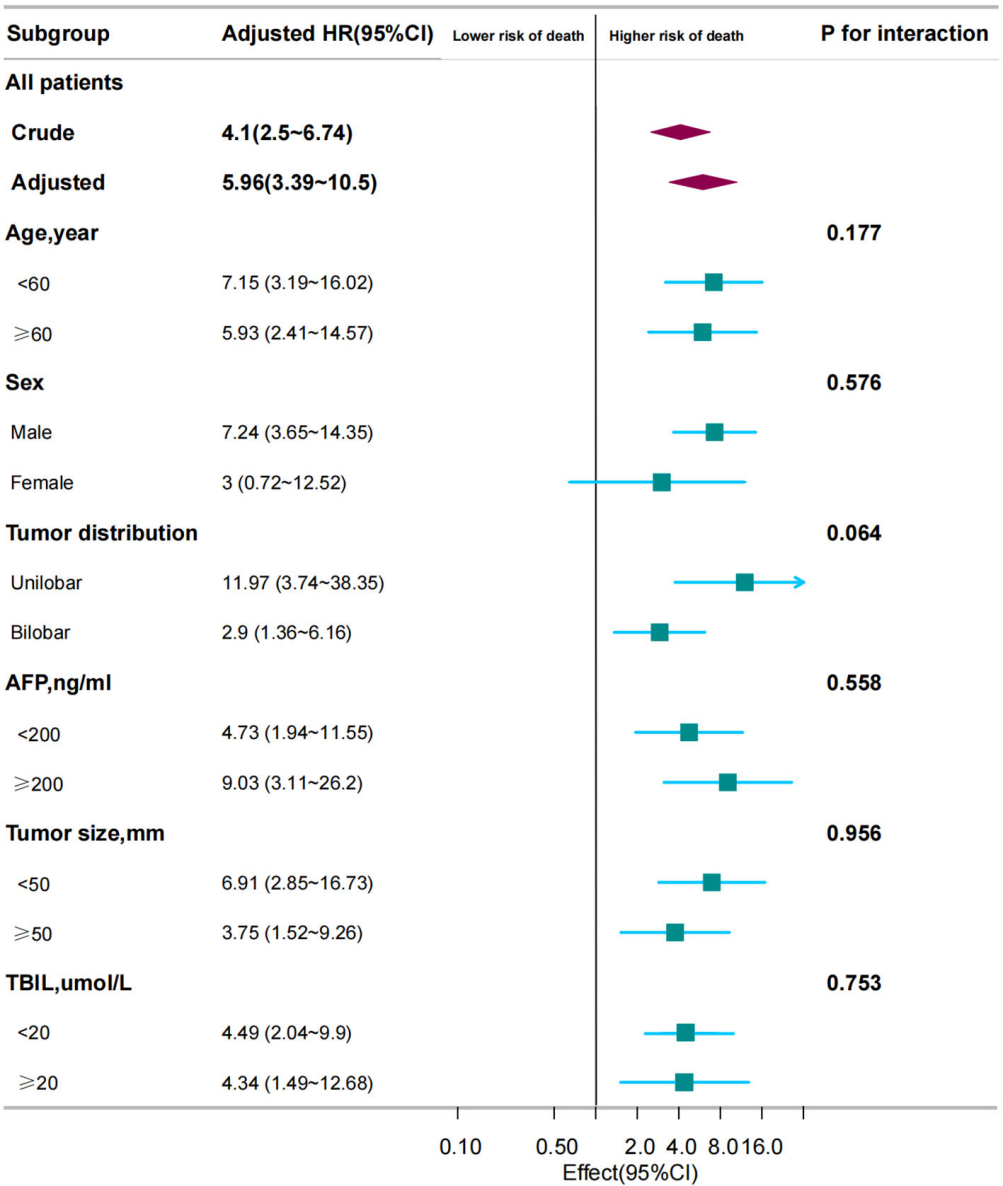
Association between TACE refractoriness and survival in different subgroups. The adjusted HR for TACE refractoriness across all patients was 5.96 (95% CI: 3.39–10.5, *P* < 0.001). Subgroup analyses show that TACE refractoriness significantly impacted OS across different ages, sexes, and tumor distributions, size and number. These findings emphasize the consistent and strong influence of TACE refractoriness on survival across various patient subgroups. HR < 1 indicates that the “TACE refractoriness group” has a lower risk of death compared to the “non-TACE refractoriness group”. HR ≥ 1 indicates that the “TACE refractoriness group” has a higher risk of death compared to the “non-TACE refractoriness group”.

Subgroup analyses were performed to examine the association between TACE-Refractoriness and OS across different patient subgroups. The results are presented in **Figure 3**. TACE-Refractoriness was significantly associated with an increased risk of death across most subgroups. For patients aged <60 years and ≥60 years, HRs were 7.15 (95% CI: 3.19–16.02) and 5.93 (95% CI: 2.41–14.57), respectively. In patients with unilobar and bilobar tumor distributions, HRs were 11.97 (95% CI: 3.74–38.35) and 2.9 (95% CI: 1.36–6.16), respectively. Stratified analyses by tumor size (<50 mm *vs*. ≥50 mm), AFP levels (<200 ng/mL *vs*. ≥200 ng/mL), and TBIL levels (<20 µmol/L *vs*. ≥20 µmol/L) yielded similar findings, with HRs exceeding 2 in all strata. However, in the female subgroup, the association between TACE-Refractoriness and OS was not statistically significant (HR = 3.0, 95% CI: 0.72–12.52).

## 4 Discussion

This study evaluated the prognostic impact of TACE-Refractoriness in patients with BCLC stage B HCC. Using a large, multicenter dataset and PSM, we found that TACE-Refractoriness is strongly associated with poor OS. Kaplan-Meier survival analyses demonstrated a significant survival disadvantage in TACE-Refractory patients both before and after matching. Multivariate Cox regression further confirmed this association, with hazard ratios consistently indicating a markedly increased risk of death after adjusting for key clinical and tumor-related covariates. These findings highlight the critical role of TACE-Refractoriness as an independent prognostic factor in BCLC stage B HCC.

The concept of TACE-refractoriness has evolved significantly over the past decade, reflecting the need for better stratification of patients undergoing TACE. Previous definitions of TACE-refractoriness, such as those proposed by the JSH and the EASL, have provided valuable frameworks for clinical decision-making (6–8). However, these definitions often lacked specificity for the Chinese population, where HCC presents with unique epidemiological characteristics. The CCI definition, which was developed in response to these challenges, integrates standardized and refined TACE procedures with disease progression assessments based on mRECIST criteria (9). Our study supports the robustness of this definition and demonstrates its relevance in predicting survival outcomes in Chinese HCC patients.

The prognostic significance of TACE-refractoriness has been underscored in several recent studies, which have consistently shown that patients who develop refractoriness to TACE experience significantly worse survival outcomes compared to those who continue to respond to treatment (19, 20). For instance, a study by Arizumi et al. (19) found that TACE-refractoriness, defined according to the JSH criteria, was associated with a median OS of only 11.2 months, compared to 31.2 months in non-refractory patients. Similarly, Yang et al. (21) reported that early TACE-refractoriness leads to significantly shorter OS compared to non-refractory patients. Our findings align with these studies, demonstrating a strong association between TACE-refractoriness and reduced OS. Furthermore, Hung et al. found that TACE-refractoriness, particularly when detected early, is linked to adverse survival outcomes, further supporting the importance of early identification and intervention strategies in managing HCC. However, there are notable differences between our results and those of earlier research. For instance, while Wang et al. (22) developed a predictive model for early TACE-refractoriness, they did not find as strong an association between TACE-refractoriness and survival as observed in our study. This discrepancy may be attributed to differences in patient populations. Additionally, our subgroup analyses showed consistent associations between TACE-Refractoriness and poor OS across most strata. However, in the female subgroup, the survival difference was not statistically significant. This may be attributed to the smaller sample size of female patients in the matched dataset, which limits the power to detect significant differences. Future studies with larger and more diverse populations are necessary to validate these findings and explore sex-based differences in outcomes. In particular, the impact of hepatitis B virus (HBV) and hepatitis C virus (HCV) related disease patterns in the Chinese population may have amplified the observed survival differences compared to studies in Western cohorts dominated by non-viral etiologies.

The role of alternative therapies in TACE-refractory patients has gained increasing importance as the limitations of repeated TACE procedures have become evident. The advent of systemic therapies, particularly tyrosine kinase inhibitors (TKIs) and immune checkpoint inhibitors (ICIs), offers new options for patients who are no longer candidates for TACE (6, 23). In the TACTICS trial, for example, the combination of TACE with sorafenib, a TKI, significantly prolonged progression-free survival (PFS) compared to TACE alone (6). Similarly, the IMbrave150 trial demonstrated that the combination of atezolizumab and bevacizumab significantly improved OS in patients with unresectable HCC, offering a promising alternative for those with TACE-refractoriness (23). Our study underscores the need for timely transition to these systemic therapies when TACE-refractoriness is identified, as continuing ineffective TACE can lead to further deterioration in liver function and poor survival outcomes.

Another important consideration is the timing of transition from TACE to systemic therapy or other treatment modalities. While the CCI criteria provide a clear framework for defining TACE-refractoriness, the optimal timing for switching therapies remains debated. Early studies suggested that continuing TACE beyond the point of refractoriness could be detrimental, leading to worsened liver function and reduced eligibility for subsequent treatments (6, 20). More recent evidence supports a proactive approach, where patients are evaluated for TACE-refractoriness after each procedure, and those showing signs of refractoriness are promptly transitioned to systemic therapy or clinical trials (2). Our findings align with this approach suggest that early identification and intervention are crucial for improving survival outcomes in TACE-refractory patients.

Despite the strengths of our study, including the use of a large, multicenter cohort and a robust propensity score-matched analysis, there are several limitations that should be acknowledged. First, as a secondary analysis based on a Chinese patient cohort, the findings may not be directly generalizable to populations in other regions where etiological factors such as HCV or non-alcoholic steatohepatitis are more prevalent. For instance, Fox et al. (16) demonstrated that prognostic factors for HCC can vary significantly across geographic regions, particularly in Western populations with a higher prevalence of HCV infection. Second, recurrence-free survival data were unavailable in the original dataset, which limited the scope of survival analysis to OS. This limitation is consistent with other retrospective studies, such as Beumer et al. (24), which emphasized the importance of integrating both OS and recurrence-free survival data for comprehensive prognostic assessments. Third, potential confounding from subsequent therapies after TACE, such as targeted therapies or ablative treatments, could not be fully accounted for due to the lack of detailed treatment information. Similar challenges were highlighted by Kudo M et al. (25), who noted that the heterogeneity in post-TACE management significantly affects outcomes and complicates analysis. Fourth, missing data for certain covariates in the original dataset required the use of multiple imputation to ensure the completeness of the dataset. While this approach minimizes bias and allows for robust statistical analysis, it cannot fully substitute for complete datasets and may introduce residual uncertainty, as also noted by Rubin DB et al. (26). Finally, the retrospective nature of this study inherently introduces potential biases and missing data, which were minimized through robust statistical techniques but cannot be entirely eliminated. Zhong et al. (27) stressed that such biases are common in retrospective studies and underscore the need for prospective validation. These limitations highlight the need for prospective studies in diverse populations and with comprehensive treatment data.

## 5 Conclusion

TACE-Refractoriness is significantly associated with poor overall survival in BCLC stage B HCC patients. This study demonstrates the importance of early identification and timely intervention in TACE-Refractory patients. Future research should validate these findings in diverse populations and explore alternative therapeutic strategies.

## Data Availability

The data utilized in this study are openly accessible and have been sourced from the Dryad Digital Repository database. These datasets can be found at https://datadryad.org/under the DOI: 10.5061/dryad.pd44k8r.
